# Proof-of-Concept Randomized Controlled Study of Cognition Effects of the Proprietary Extract *Sceletium tortuosum* (Zembrin) Targeting Phosphodiesterase-4 in Cognitively Healthy Subjects: Implications for Alzheimer's Dementia

**DOI:** 10.1155/2014/682014

**Published:** 2014-10-19

**Authors:** Simon Chiu, Nigel Gericke, Michel Farina-Woodbury, Vladimir Badmaev, Hana Raheb, Kristen Terpstra, Joalex Antongiorgi, Yves Bureau, Zack Cernovsky, Jirui Hou, Veronica Sanchez, Marissa Williams, John Copen, Mariwan Husni, Liz Goble

**Affiliations:** ^1^Lawson Health Research Institute, Lab FB125, 268 Grosvenor Street, London, ON, Canada N6A 4V2; ^2^Department of Psychiatry, University of Western Ontario, London, ON, Canada N6A 3K7; ^3^Medical and Scientific of HG & H Pharmaceuticals (Pty) Ltd., 193 Bryanston Drive, Bryanston 2192, South Africa; ^4^Department of Psychiatry, School of Medicine, University of Puerto Rico, San Juan, PR 00936-5067, USA; ^5^Medical and Scientific Affairs, P L Thomas & Co., Inc., Morristown, NJ 07960, USA; ^6^Department of Psychiatry, Island Medical Program, University of Victoria, University British Columbia Extended Medical Campus, Victoria, BC, Canada V8W 2Y2; ^7^Department of Psychiatry, Northern Ontario School of Medicine, Thunder Bay, ON, Canada P7B 5E1

## Abstract

*Introduction*. Converging evidence suggests that PDE-4 (phosphodiesterase subtype 4) plays a crucial role in regulating cognition via the PDE-4-cAMP cascade signaling involving phosphorylated cAMP response element binding protein (CREB). *Objective*. The primary endpoint was to examine the neurocognitive effects of extract *Sceletium tortuosum* (Zembrin) and to assess the safety and tolerability of Zembrin in cognitively healthy control subjects. *Method*. We chose the randomized double-blind placebo-controlled cross-over design in our study. We randomized normal healthy subjects (total *n* = 21) to receive either 25 mg capsule Zembrin or placebo capsule once daily for 3 weeks, in a randomized placebo-controlled 3-week cross-over design. We administered battery of neuropsychological tests: CNS Vital Signs and Hamilton depression rating scale (HAM-D) at baseline and regular intervals and monitored side effects with treatment emergent adverse events scale. *Results*. 21 subjects (mean age: 54.6 years ± 6.0 yrs; male/female ratio: 9/12) entered the study. Zembrin at 25 mg daily dosage significantly improved cognitive set flexibility (*P* < 0.032) and executive function (*P* < 0.022), compared with the placebo group. Positive changes in mood and sleep were found. Zembrin was well tolerated. *Conclusion*. The promising cognitive enhancing effects of Zembrin likely implicate the PDE-4-cAMP-CREB cascade, a novel drug target in the potential treatment of early Alzheimer's dementia. This trial is registered with ClinicalTrials.gov NCT01805518.

## 1. Introduction

With the global increase in life expectancy, productive cognitive aging focuses on longevity and enhancing quality of life, reducing age-related cognitive decline, and preventing Alzheimer's dementia [1]. Preventive proactive health strategies explore multiple tools and resources to boost “cognitive reserve” and to consolidate “cognitive maintenance” [2, 3]. Recent research advances in aging and Alzheimer's dementia (AD) have provided evidence to define the clinical stages of AD into three stages: (1) preclinical; (2) mild-cognitive impairment (MCI), and (3) AD [4]. Neuroimaging positron emission tomography (PET) approach to label amyloid-*β*1-42 (A*β*1-42) peptide showed that in the preclinical stage A*β*1-42 started to accumulate in the vulnerable brains of individuals 1 to 2 decades prior to cognitive impairment [5]. The disease burden of AD can be present before any symptoms appear. This finding opens up new avenues of proactive interventions at the asymptomatic stage with the objective to “postpone the onset, reduce the risks of, or completely prevent the clinical stages of AD” [6]. In this respect dietary interventions through hitting multiple targets for AD: amyloid-A*β*1-42 aggregation, neuroinflammation, synaptic plasticity, and tau (microtubule-associated protein) phosphorylation, can offer unique advantages to attenuate the self-propagating course of AD and to prevent the transition from MCI to early AD [7].

There has been growing interest in the crucial role of intracellular second messengers: cAMP and cGMP, in regulating age-associated gene expression and cognitive decline [8]. The dual second messengers induce signal cascades involved in neuronal survival, apoptosis, and synaptic plasticity and neural connectivity. The cAMP-response element binding protein (CREB) appears to orchestrate the signal transduction from gene activation to effector responses of cognition. The aging process is linked to differential changes in gene expression of PDE subtypes [9]. In the rodent species, PDE-4 gene knockout resulted in memory improvement in the rodent species [10]. Rolipram, the prototypal PDE-4 antagonist, reversed A*β*25-35 and A*β*1-40 peptide-induced memory deficits in the Morris-water maze test (MWM) and passive avoidance tasks [11]. Results from translational research studies raise the issue of whether consumption of PDE-driven nutraceuticals in middle-aged adults can provide neuroprotective effects in preventing or delaying the onset of cognitive decline.

Promising findings have been reported on the cognitive benefits of early intervention with nutraceuticals targeting PDE-4-cAMP-CREB signaling [12, 13]. Resveratrol isolated from grapes is present in the red wine and has been shown to modify cognition through the PDE-4-cAMP metabolic pathway [14]. Caffeine (1,3,5-trimethylxanthine), widely consumed as coffee in the world, targets both PDE subtypes 4 and 5 (PDE-4, PDE-5) and adenosine-2 receptor [15]. Epidemiological studies indicate that coffee/caffeine consumption is likely to be associated with a reduced risk of AD [16, 17]. In the prospective Cardiovascular Risk Factors, Aging, and Dementia (CAIDE) study, daily coffee drinking totaling 3–5 cups per day at midlife reduced AD risk by about 65% at late life [18].

**Figure 1:**
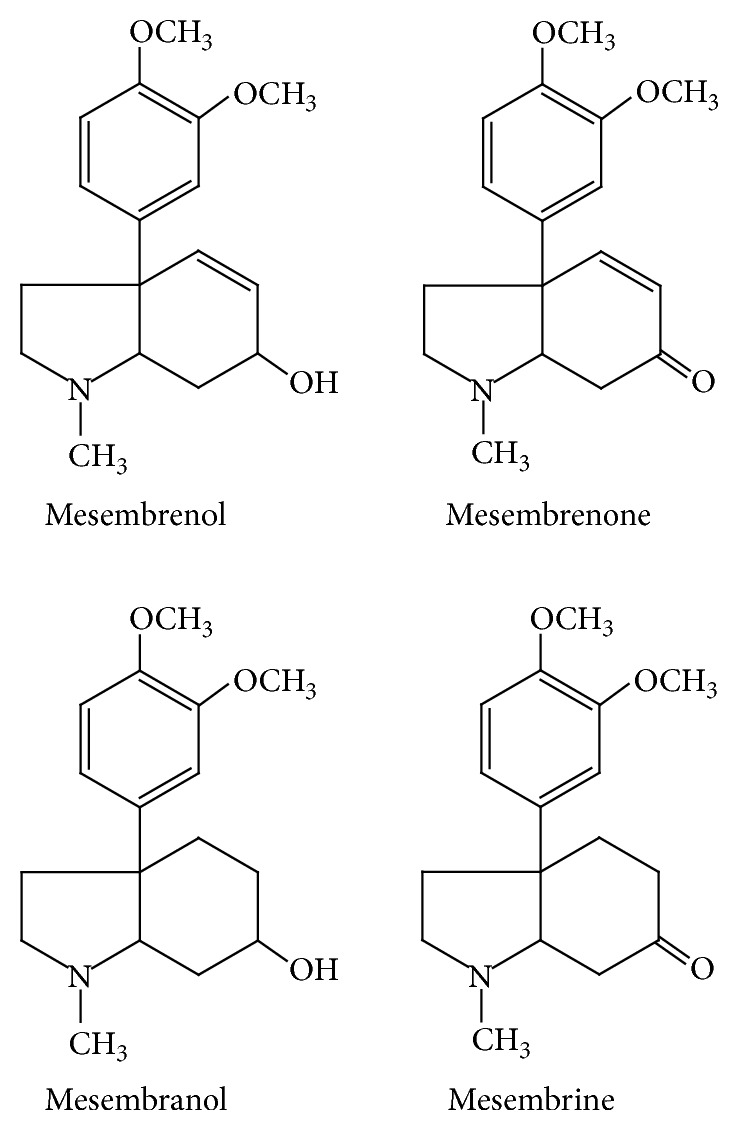
Chemical structures of alkaloids isolated from* Sceletium tortuosum*: mesembrine, mesembrenol, mesembrenone, and mesembranol.

In the recombinant PDE4* in vitro* assay,* Sceletium tortuosum* (Zembrin) selectively inhibited PDE-4 with IC50 (inhibitory constant) value of 8.5 *μ*g/mL. Mesembrenone, a major alkaloid isolated from the extract (Figure 1), was active in inhibiting PDE4 with an IC50 value of <1 *μ*M [19]. The* in vitro* findings are consistent with Zembrin in MWM test [20].

In our proof-of-concept randomized placebo-controlled study, we hypothesize that Zembrin enhances cognition in healthy subjects. Previously, a RCT trial reported that daily oral dosage of 25 mg for 3 months was safe and well tolerated [21]. Our study demonstrated for the first time that Zembrin improved executive function and cognitive flexibility in healthy control subjects.

## 2. Materials and Methods

### 2.1. Study Design

The pilot study was a randomized placebo-controlled cross-over study using a fixed oral dosage of 25 mg extract* Sceletium tortuosum* (Zembrin) taken once daily. The study was conducted at a single center in Puerto Rico (a territory administered by the USA). The duration of the study was 9 weeks.

### 2.2. Study Product

Extract* Sceletium tortuosum* (Zembrin) was manufactured according to European Union Good Manufacturing Practice (GMP). The extract was in the form of a fine dry powder with the dry plant material : extract ratio of 2 : 1, standardized to a total alkaloid content for the four main Sceletium alkaloids (mesembrenone, mesembrenol, mesembrine, and mesembranol) of 0.4%. The contents of the four alkaloids were quantified using high pressure liquid chromatography (HPLC) analysis against validated analytical reference standards. Each active opaque white gelatin capsule, lot number NG022, contained 25 mg of extract* Sceletium tortuosum* (Zembrin) lot number SCE0411-1605, equivalent to 50 mg of a cultivated selection of traditionally used* Sceletium tortuosum *plant material. Identical-looking placebo capsules, lot number NG009, contained no herbal extract. Placebo and Zebrine capsules were identical in size, transparency, color, and taste.

### 2.3. Subject Recruitment

Potential research subjects aged 45 to 65 years old of either gender were recruited at a single site in Puerto Rico (US) through advertisement. They were required to undergo screening with the structured clinical interview for psychiatric disorders: Mini-Neuropsychiatric Interview MINI-Plus to identify the presence of any DSM IV-R psychiatric disorders [22]. Subjects were excluded if they had any significant and untreated medical disorders, including severe uncontrolled or marginal glycemic controlled diabetes mellitus, recent myocardial ischemia or infarction, unstable angina, uncontrolled hypertension, renal failure and serious renal diseases, chronic active hepatitis, acute hepatitis, cirrhosis of liver, AIDS, malignancy, and neurological disorders: epilepsy, recent cerebrovascular disease, and recent traumatic brain injury. Subjects with active suicidal risk were also excluded. Pregnant females and subjects with HAM-D (Hamilton depression) [23] score >/−8 and/or body mass index BMI >/−30.0 were also excluded.

### 2.4. Study Protocol

At baseline, subjects were required to undergo physical examination, blood chemistry, and urine toxicological screen and 12-lead EKG, along with neurocognitive assessment using the computerized battery of validated neuropsychological tests named CNS Vital Signs. We had privileged access to the CNS Vital Signs through an academic-research contract with CNS Vital Signs Inc., USA [24]. The test battery was administered under standardized conditions by qualified personnel. The battery of neuropsychological tests was developed to measure the changes in 9 cognitive domains: composite memory, verbal memory, visual memory, processing speed, executive function, psychomotor speed, reaction, complex attention, and cognitive flexibility. The subjects were also administered HAM-D and treatment emergent adverse events at face-to-face interview with research clinicians experienced in clinical trials. Subjects were randomized by the procedure of computerized random numbers to either one of the two arms: (1) the active investigational product arm received extract* Sceletium tortuosum* (Zembrin) 25 mg capsule (abbreviated Zembrin group); (2) the placebo arm received placebo capsule (abbreviated placebo group). During the first 3-week phase of the study blinded to both the researchers and the subjects, the participants randomized to the Zembrin group took a 25 mg capsule of Zembrin once daily orally whereas the placebo group took a placebo capsule once a day orally. At the end of the first 3-week phase, the subjects were allowed a 3-week washout, during which time no capsules were ingested by either group. During the second 3-week switch-over phase, the Zembrin group took a placebo capsule once a day orally and the placebo group took the 25 mg Zembrin capsule once daily orally. During the entire 9-week study period, vital signs (blood pressure and pulse) and body weight were monitored. CNS Vital SignR, HAM-D, and treatment emergent adverse events were administered at baseline and the end of weeks 3, 6, and 9. The study was fully approved by Aspire Inc. (CA, USA), a community institutional research ethics board granting approval and monitoring clinical trials. Research subjects participated in the study only after they were fully explained the protocol and signed the informed consent form.

### 2.5. Statistics

We used the one-way analysis of variance (ANOVA) followed by the post hoc Tukey HSD test to examine the difference in changes in neurocognitive data from Zembrin-treated group and the placebo group, as compared with baseline. The level of significance was set at *P* < 0.05 for two-tailed paired* t*-test and nonpaired* t*-test. Cohen's* d* statistics was used to evaluate the treatment effect sizes of the placebo and Zembrin groups. For Cohen's* d* effect size, a positive value > 0.0 indicates that Zembrin treatment is better than placebo, whereas a negative value < 0.00 shows that the placebo is better than Zembrin, under the conditions of the study protocol. For Cohen's* d*, the higher the value of Cohen's* d*, the larger the treatment effect. Chi-square statistics and Kendall's tau were used to determine the frequency of adverse events.

## 3. Results

### 3.1. Baseline Clinic-Demographics

A total of 21 subjects (male to female ratio, 9/12) were recruited at the single research center in PR (US). The mean age was 54.6 years (range 45 to 61 years, SD = 6.0). Two subjects dropped out during the placebo phase and three subjects dropped out during the Zembrin phase. During the screening phase, healthy subjects with neither major medical disorders nor DSM IV-R psychiatric disorders signed the consent form after the study protocol was fully explained to them. Completer-analysis criteria were used in analyzing the data.

### 3.2. Efficacy

**Figure 2:**
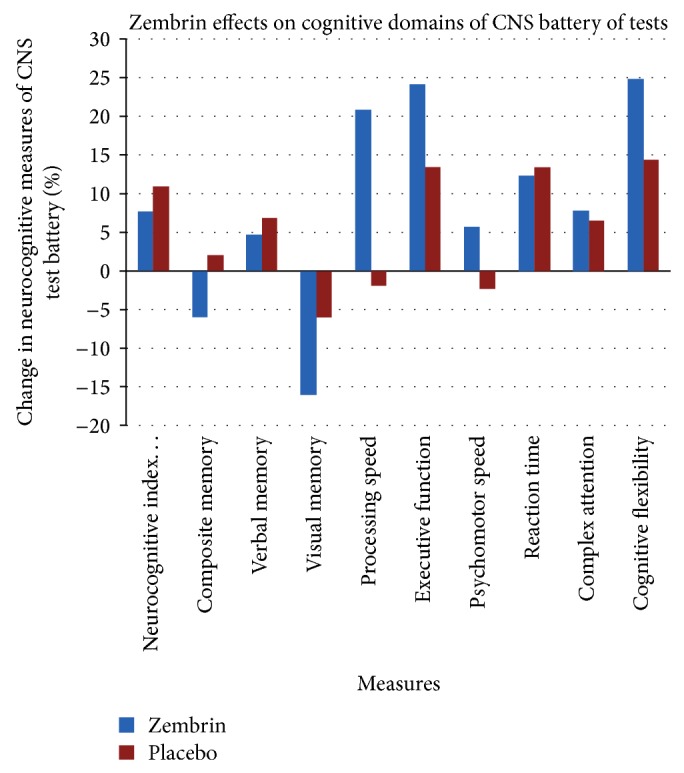
Cognitive effects of Zembrin extract in healthy subjects: Zembrin-treated subjects and placebo-treated subjects were administered CNS Vital SignR battery of neuropsychological tests at baseline and at the end of the treatment period. Cognitive domains were derived from CNS Vital SignR tests.

The primary efficacy endpoint was the change in eight cognitive function domains and the aggregate average of the neurocognitive measure defined as neurocognitive index as measured with the CNS Vital SignR test. The data were analyzed statistically by one-way ANOVA with 3 groups of data as shown in Table 1. We compared the changes in the overall performance score named as neurocognitive index as the summed total score of the 9 cognitive domains: composite memory, verbal memory, visual memory, processing speed, executive function, psychomotor speed, reaction, complex attention, and cognitive flexibility (Figure 2). For each specific neurocognitive measure, we also examined whether the Zembrin group differed from the placebo group. We evaluated the changes in cognitive score at baseline with the score at the end of the three weeks of placebo or Zembrin period. No cross-over effect was found for either the placebo or the extract groups.

As shown in Table 1 and Figure 2, the ANOVA analysis followed by Tukey HSD post hoc test indicated that the mean neurocognitive index and score for each separate domain differed between the Zembrin phase and the placebo phase. Zembrin significantly improved cognitive flexibility (*P* < 0.022) and executive function (*P* < 0.032) as compared with placebo. Zembrin improved processing speed, psychomotor speed, and complex attention, but ANOVA analysis failed to find a statistical significance between the groups in composite memory, verbal memory, and visual memory. Both the placebo and Zembrin groups showed negative change in the domain of visual memory. In a separate analysis, we found that the final visual memory score differed only minimally from the baseline value. Furthermore, the baseline visual memory score of placebo group remained unchanged at the end of the 3rd week period (39.4 (SD = 32.0) versus average 39.6 (SD = 29.0)). We interpret the findings to be related to practice effect. The cohort of cognitively normal subjects may be more sensitive to the negative practice effects.

Regarding the neurocognitive data, Cohen's* d* statistics estimate of effect size favored Zembrin group over the placebo group. We found robust Cohen's* d* effect size for two cognitive domains: 1.47 for cognitive flexibility and 1.49 for executive function. For processing speed, a strong Cohn's effect size was found: 2.88, whereas for psychomotor speed Cohen's* d* was 0.95 and a small effect in the domain of complex attention: 0.16.

The subjective ratings of generalized well-being and positive mood states in our study agreed well with a recent safety and tolerability study on extract* Sceletium tortuosum* (Zembrin) in healthy subjects who reported “uplifted spirits” and better coping with stress and depressing events [21]. The baseline HAM-D scores in both groups were <6.0 at the start of the study. As shown in Table 2, for the Zembrin group the change from the baseline value of 2.17 (SD = 1.9) to 1.22 (SD = 1.9) after 3 weeks represented a 26.9% change. For the placebo group, the baseline HAM-D was 1.95 (SD = 2.1) and changed to 1.68 (SD = 1.5) after 3 weeks on placebo, representing a 13.8% decrease in HAM-D. In view of the substantial variation, the difference failed to reach statistical significance.

We observed that subjects taking Zembrin reported improvement in the subjective quality of their sleep on the HAM-D subscale. While none of the subjects had a history of insomnia, the data showed that Zembrin had a positive effect on onset of sleep, as compared with the placebo group. We observed that the mean rating of sleep onset at baseline, 0.76 (SD = 1.14), changed to 0.43 (SD = 0.93) at week 3 (paired* t* test,* t* = 2.09, df = 20, *P* = 0.049, 2-tailed). No significant change was noted in the placebo-treated group regarding the variable: sleep-onset difficulty: *P* = 0.606, pre- and posttreatment* t*-test. In this study, Zembrin exerted an overall improvement in the quality of sleep in a normal community sample. Our results corroborated the findings from a recent safety and tolerability study of extract* Sceletium tortuosum* (Zembrin) on sleep quality [21].

### 3.3. Safety and Tolerability

Extract* Sceletium tortuosum* (Zembrin) was safe and well tolerated. There were no changes in blood pressure, pulse, temperature, and weight in either the Zembrin group or the placebo group (Table 3). Regarding the treatment emergent adverse events (Table 4) the Zembrin group reported transient gastrointestinal discomfort (9.5%) which resolved spontaneously, and the clinician did not ascribe a direct link to the Zembrin. This study agrees with a recent published RCT study reporting the placebo group complained of more frequent adverse events than the* Sceletium tortuosum* (Zembrin) group [21]. The incidence of treatment emergent adverse events (TEAE) classed as “mild” and “moderate” was quite low. The common side effects in the placebo group consisted of mild skin irritation (10%), appetite decrease (10%) and weight loss (15%), and interrupted sleep (10%).

We used Kendall's tau *c* coefficient (a nonparametric statistical measure of association between two measured quantities and unaffected by deviations from the normal data distribution) to analyze the direction of changes in the frequency and intensity of the complaints and symptoms as listed in the treatment emergent adverse events (TEAE) scale. The tau *c* coefficients are interpreted as follows: 0 indicates no association, 0–0.1 weak association, 0.1–0.3 moderate associations, and 0.3–1.0 strong association. We determined the tau *c* coefficients for the baseline to end of 3rd week treatment with Zembrin and with placebo. The findings indicated the Zembrin group showed selective improvement in the following subcategories: irritability (tau  *c* = 0.33), drowsiness (tau  *c* = 0.30), memory problems (tau  *c* = 0.25), anxiety (tau  *c* = 0.24), confusion (tau  *c* = 0.17), skin irritation (tau  *c* = 0.16), chest pain (tau  *c* = 0.15), stool discoloration (tau  *c* = 0.12), and poor hearing (tau  *c* = 0.11). For the placebo group, there was an association with decrease in appetite (tau  *c* = 0.20) and weight gain (tau  *c* = 0.26).

## 4. Discussion

Our study demonstrated for the first time in healthy subjects Zembrin extract in selectively targeting PDE-4 significantly enhanced executive function and cognitive flexibility. Zembrin was highly tolerated. Executive function encompasses higher-order cognitive processes such as working memory, attention control, response inhibition, and concept formulation and is thought to be primarily driven by the prefrontal cortex. Cognitive intact individuals can modify behavioral repertoire and cognitive set in response to changing environmental cues and contexts and hence can streamline the activities of daily living (ADL) [25, 26]. In general, executive functions are crucial for maintaining the cognitive homeostasis of the individuals in order to meet the demands of daily living.

There is a growing body of evidence to indicate that impairment in executive functioning is present at the very earliest stage of Alzheimer's dementia before marked memory deficits appear. In a large cohort study of healthy control along with early Alzheimer's dementia (AD) [27] and mild cognitive impairment (MCI) (*n* = 793) subjects, multiple regression analysis showed a significant relation between executive function and impairment in instrumental activities of daily living (IADL). In the preclinical stage of AD, the two measures of executive function: color word interference test (CWIT) and verbal fluency (VF) predicted decline in cognitive set switching [28]. Two specific measures of executive function, Stroop test and sematic fluency test, were found to correlate with reduced glucose utilization as examined with PET ligand, [18F] fluorodeoxyglucose (FDG) [29]. Decline in executive function has been localized in the left lateral prefrontal cortex, the crucial brain region related to the triad components of the cognitive control: working memory, task switching, and inhibitory control [30, 31].

The cognitive effects of Zembrin are consistent with the* in vitro* structure-activity relationship analysis showing cognition is selectively mediated via the long isoform of PDE-4D3 [32]. In the PDE assay system using the human recombinant PDE-4B, mesembreonone was 17 times more potent than mesembrine and 34 times more active than mesembrenol in inhibiting PDE-4B binding [19, 21, 33]. The IC50 values for mesembrenone were 470 nM, followed by mesembrine 7800 nM and mesembrenol 10,000 nM. In the macaque monkey species, rolipram, the prototypal PDE-4 antagonist, has been found to improve object retrieval performance [34]. Zembrin distinguishes from other PDE-4 agonists in the absence of gastrointestinal side effects [35].

**Figure 3:**
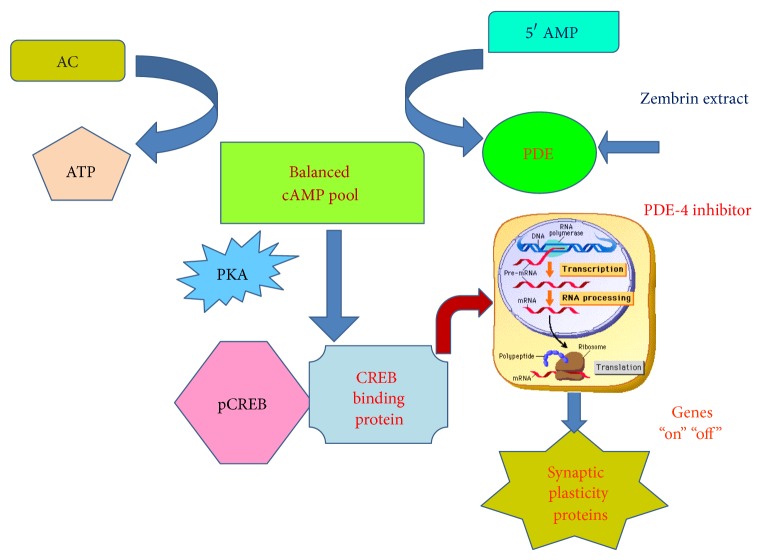
PDE-4/cAMP/CREB cascade in cognition: bidirectional regulation of cyclicAMP (cAMP) homeostasis is achieved through adenylate cyclase (AD) and phosphodiesterase (PDE) subtype 4. cAMP is activated via either PDE-4 inhibition or hormone/neurotransmitter-stimulated adenylate cyclase (AD). The cAMP-dependent protein kinase A (PKA), once activated through allosteric site, can phosphorylate cAMP response element binding protein (CREB) to form phosphorylated CREB: pCREB. pCREB associates with transcription coactivator, CREB binding protein to initiate transcription and translation. The CREB-mediated gene expression contributes towards long- and short-term memory and synaptic plasticity. Zembrin extract modulates PDE-4 and participates in PDe-4/cAMP/CREB cascade in cognition. The CREB-linked gene expression has been shown to be impaired in AD model. ATP: adenosine triphosphate; cAMP: cyclic adenosine monophosphate; adenylate cyclase: AC; 5′AMP: 5′ adenosine monophosphate; CREB: cAMP response element binding protein; pCREB: phosphorylated CERB; PKA: phosphokinase A.

We propose the model of PDE-coupled cAMP cascade to explain the cognitive effects of Zembrin (Figure 3). Zembrin is behaving as the putative PDE-4 allosteric modulator interacting with PDE-4-coupled system. The synaptic spinoffs in aging are seen in changes in modulating the downstream CREB-mediated effector responses comprising neurogenesis, synapse remodeling, and neuron-microglia reciprocal regulation [35].

The results of our study on the cognitive enhancing effects of Zembrin extract, however, may not be interpreted solely in the context of PDE-4 modulation. The family of mesembrine alkaloids from Zembrin exhibit serotonergic properties in competing for serotonin transporter site* in vitro* [23, 24]. Serotonin is known to play a central role in decision processing and attentional set shifting [36]. Very recently, citalopram, the selective serotonin reuptake inhibitor (SSRI), has been shown to reduce the CSF amyloid A*β* load in both normal human subjects and in transgenic mice [37]. Hence, serotonin signaling may synergize with PDE-coupled camp cascade in modulating age-related cognition changes [38].

Our initial surreptitious findings of Zembrin in bringing about positive changes in sleep and mood are only preliminary and have to be interpreted with caution. A very recent functional magnetic resonance imaging fMRI study found region-specific brain activation effects of Zembrin [39]. Acute administration of Zembrin extract in normal healthy subjects dampened amygdala reactivity to fearful faces under low perceptual load conditions, along with reduced coupling of amygdala-hypothalamus as shown by follow-up connectivity analysis on the emotion-matching task [39]. Nevertheless, the emerging body of evidence suggests that PDE-4-cAMP-CREB signal cascade plays a crucial role in regulating sleep-wakefulness cycle and affective regulation. In sleep deprivation model, the C57BL/6J mice when sleep deprived for 5 hours showed selective impairment in cAMP/phosphokinase A-dependent synaptic plasticity which is reflected in the long-term potentiation (LTP) paradigm, in the hippocampus [40]. Genetic knock-down of PDE-4D enzyme: PDE4d (−/−) mice displayed an antidepressant phenotype in two behavioral models: the Porsolt forced swim test and the acoustic startle response [41]. No adverse changes were noted in the sensorimotor gating deficits. Taken together, the potential anxiolytic/antidepressant and sleep-promoting properties of Zembrin warrant further investigations in the elderly.

## 5. Conclusion

Despite the methodological limitations of small sample size, the short treatment period, and the single dosage, we demonstrated for the first time that Zembrin enhanced cognition in the domains of executive functioning and cognitive flexibility in healthy subjects. We will extend our study to the cohort of elderly subjects to further investigate whether Zembrin can delay or reverse the course of age-related cognitive decline and AD as well as prevent the conversion from MCI to AD. Our study of the cognitive benefits of Zembrin highlights the pivotal role of nutraceuticals in ushering a new era of preclinical AD preventive program starting at midlife towards old age. We anticipate that biotechnology advances will accelerate the transformation of PDE-4 molecular signatures to a new class of promising drug candidates for the treatment of AD.

## Figures and Tables

**Table 1:** Cognitive outcome measures of Zembrin effects on neurobattery tests after 3 weeks of Zembrin treatment and placebo treatment.

Outcome measure	Mean score and SEM at baseline and after Zembrin and after placebo treatment						
CNS Vital Signs Key domains:	Baseline	After 3 weeks Zembrin		After 3 weeks of placebo		*F* values	*P* values
		(*N* = 17)	[% change]	(*N* = 18)	[% change]		
Neurocognitive index (NCI)	36.3 (4.5)	43.9 (6.8)	[7.6]	47.2 (7.7)	[10.9]	*F* = .992	*P* = 0.376
Composite memory	38.9 (5.3)	32.9 (8.5)	[−6.0]	41.0 (9.0)	[2.1]	*F* = .269	*P* = 0.765
Verbal memory	36.9 (5.1)	41.6 (7.7)	[4.7]	43.7 (8.9)	[6.8]	*F* = .302	*P* = 0.740
Visual memory	45.6 (5.5)	29.5 (8.6)	[−16.1]	39.6 (7.2)	[−6.0]	*F* = 1.403	*P* = 0.253
Processing speed	56.7 (5.7)	77.4 (6.8)	[20.7]	54.7 (8.8)	[−2.0]	*F* = 2.557	*P* = 0.085
Executive function	36.8 (5.4)	60.8 (6.6)	[24.0]	50.1 (7.7)	[13.3]	^*^ *F* = 3.603	^*^ *P* = 0.032
Psychomotor speed	54.8 (5.6)	60.4 (8.2)	[5.6]	52.4 (8.5)	[−2.4]	*F* = .252	*P* = 0.778
Reaction time	45.8 (5.1)	58.1 (6.3)	[12.3]	59.1 (6.8)	[13.3]	*F* = 1.686	*P* = 0.193
Complex attention	38.5 (5.3)	46.2 (7.8)	[7.7]	44.9 (8.5)	[6.4]	*F* = .407	*P* = 0.667
Cognitive flexibility	**35.4 (5.3)**	**60.2 (6.5)**	**[24.8]**	**49.7 (7.5)**	**[14.3]**	*F* = 4.016	^*^ *P* = 0.022

^*^Statistically different at *P* < 0.05 two-tailed.

**Table 2:** Zembrin extract effect on HAM-D in healthy control subjects.

	Average HAM-D	SD	*P* value 1-tailed	Effect size: % change
Before Zembrin (*N* = 18)	1.67	1.9	*P* = 0.075	26.9% decrease on HAM-D
After Zembrin (*N* = 18)	1.22	1.9		

Before placebo (*N* = 19)	1.95	2.1	*P* = 0.289	13.8% decrease on HAM-D
After placebo (*N* = 19)	1.68	1.5		

**Table 3:** Zembrin extract on vital signs in healthy control subjects.

	Zembrin: means (SDs); *N* = 20			Placebo: means (SDs); *N* = 20		
	Before	After	*P* value	Before	After	*P* value
Systolic BP	115.0 (13.2)	116.7 (15.8)	ns	117.5 (17.4)	116.3 (13.8)	ns
Diastolic BP	72.3 (8.7)	73.4 (10.4)	ns	76.0 (9.4)	74.5 (7.5)	ns
Pulse	73.4 (7.2)	74.6 (7.1)	ns	74.4 (11.9)	75.7 (9.3)	ns
Temperature	36.2 (0.2)	36.1 (0.2)	ns	36.3 (0.3)	36.3 (0.3)	ns
Weight (lbs)	163.1 (26.0)	162.6 (26.7)	ns	162.7 (26.0)	163.5 (26.8)	ns

Ns: not significant at *P* < 0.05 level.

**Table 4:** Treatment emergent adverse events: Zembrin versus placebo.

Category	Signs/symptoms	Minimal = 1		Mild = 2		Moderate = 3		Severe = 4	
		Z	P	Z	P	Z	P	Z	P
Head	Headache	0	0		0	0	0	5	0
Eyes	Eye irritation	0	0	0	0	0	0	0	5
Eyes	Vision blurred	0	0	5	0	0	0	0	5
Ears	Poor hearing	5	5	0	0	0	0	0	0
Mouth	Hypersalivation	0	5	0	0	0	0	0	0
Mouth	Dental problems	0	0	0	0	5	5	0	0
Chest	Chest pain	0	0	5	0	0	0	0	0
Chest	Coughing	0	5	0	0	0	0	0	0
Gastrointestinal	GI discomfort	0	0	0	5	0	0	9.5	0
GI	Nausea	0	0	0	0	5	0	0	0
GI	Vomiting	0	0	0	0	5	0	0	0
GI	Constipation	0	0	0	0	0	0	5	0
GI	Flatulence	0	0	0	5	0	0	5	0
GI	Appetite increase	5	0	0	0	5	0	0	0
GI	Appetite decrease	0	5	0	10	0	5	0	0
GI	Weight gain	14	0	0	0	0	0	0	0
GI	Weight loss	5	10	0	15	0	0	0	0
GI	Thirst increase	0	0	5	0	5	0	0	0
GU	Increased frequency	0	0	0	5	0	5	0	0
GU	Genital discomfort	0	0	0	0	5	0	0	0
GU	Increased libido	0	0	0	0	0	5	0	0
Musculoskeletal	Muscle/bone/joint pain	5	0	0	5	0	10	0	5
MS	Rigidity (muscle)	0	0	0	0	5	0	0	0
Skin	Skin irritation	0	0	0	10	0	10	0	0
Skin	Hair problems	0	5	0	5	0	5	0	0
Psychiatric: PS	Tiredness/fatigue	0	0	5	0	0	0	5	0
PS	Overarousal	0	5	0	0	0	5	0	0
PS	Difficulty falling asleep	0	0	0	10	0	0	0	0
PS	Early morning awakenings	5	5	0	5	0	0	0	0
PS	Interrupted sleep	0	0	0	10	0	0	0	**0**
PS	Drowsiness	0	0	0	0	0	0	5	**0**
PS	Confusion	0	0	0	0	5	0	0	**0**
PS	Concentration difficulty	0	0	0	0	0	0	5	**0**
PS	Memory problems	0	5	0	0	5	0	0	**0**
PS	Depression	0	0	0	0	5	0	0	**0**
PS	Anxiety	0	5	0	0	5	0	0	**0**
PS	Irritability	**0**	**0**	**0**	**5**	**0**	**0**	**0**	**0**

NB: no life-threatening adverse events were found.

Frequency of adverse events ≥5%. Z: Zembrin treated group. P: placebo treated group.

## References

[1] Ljubuncic P., Globerson A., Reznick A. Z. (2008). Evidence-based roads to the promotion of health in old age. *Journal of Nutrition, Health and Aging*.

[2] Nyberg L., Lövdén M., Riklund K., Lindenberger U., Bäckman L. (2012). Memory aging and brain maintenance. *Trends in Cognitive Sciences*.

[3] Daffner K. R. (2010). Promoting successful cognitive aging: a comprehensive review. *Journal of Alzheimer's Disease*.

[4] Hyman B. T., Phelps C. H., Beach T. G., Bigio E. H., Cairns N. J., Carrillo M. C., Dickson D. W., Duyckaerts C., Frosch M. P., Masliah E., Mirra S. S., Nelson P. T., Schneider J. A., Thal D. R., Thies B., Trojanowski J. Q., Vinters H. V., Montine T. J. (2012). National Institute on Aging-Alzheimer's Association guidelines for the neuropathologic assessment of Alzheimer's disease. *Alzheimer's and Dementia*.

[5] Hatashita S., Yamasaki H. (2013). Diagnosed mild cognitive impairment due to Alzheimer's disease with PET biomarkers of beta amyloid and neuronal dysfunction. *PLoS ONE*.

[6] Otaegui-Arrazola A., Amiano P., Elbusto A., Urdaneta E., Martínez-Lage P. (2014). Diet, cognition, and Alzheimer's disease: food for thought. *European Journal of Nutrition*.

[7] Laditka J. N., Laditka S. B., Tait E. M., Tsulukidze M. M. (2012). Use of dietary supplements for cognitive health: results of a national survey of adults in the United States. *The American Journal of Alzheimer's Disease and other Dementias*.

[8] Benito E., Barco A. (2010). CREB's control of intrinsic and synaptic plasticity: implications for CREB-dependent memory models. *Trends in Neurosciences*.

[9] Bollen E., Prickaerts J. (2012). Phosphodiesterases in neurodegenerative disorders. *IUBMB Life*.

[10] Li Y.-F., Cheng Y.-F., Huang Y., Conti M., Wilson S. P., O'Donnell J. M., Zhang H.-T. (2011). Phosphodiesterase-4D knock-out and RNA interference-mediated knock-down enhance memory and increase hippocampal neurogenesis via increased cAMP signaling. *Journal of Neuroscience*.

[11] Cheng Y.-F., Wang C., Lin H.-B., Li Y.-F., Huang Y., Xu J.-P., Zhang H.-T. (2010). Inhibition of phosphodiesterase-4 reverses memory deficits produced by A*β*25-35 or A*β*1-40 peptide in rats. *Psychopharmacology*.

[12] Saura C. A., Valero J. (2011). The role of CREB signaling in Alzheimer's disease and other cognitive disorders. *Reviews in the Neurosciences*.

[13] Blokland A., Menniti F. S., Prickaerts J. (2012). PDE Inhibition and cognition enhancement. *Expert Opinion on Therapeutic Patents*.

[14] Chung J. H. (2012). Metabolic benefits of inhibiting cAMP-PDEs with resveratrol. *Adipocyte*.

[15] Rivera-Oliver M., Díaz-Ríos M. (2014). Using caffeine and other adenosine receptor antagonists and agonists as therapeutic tools against neurodegenerative diseases: a review. *Life Sciences*.

[16] Santos C., Costa J., Santos J., Vaz-Carneiro A., Lunet N. (2010). Caffeine intake and dementia: systematic review and meta-analysis. *Journal of Alzheimer's Disease*.

[17] Eskelinen M. H., Kivipelto M. (2010). Caffeine as a protective factor in dementia and Alzheimer's disease. *Journal of Alzheimer's Disease*.

[18] Eskelinen M. H., Ngandu T., Tuomilehto J., Soininen H., Kivipelto M. (2009). Midlife coffee and tea drinking and the risk of late-life dementia: a population-based CAIDE study. *Journal of Alzheimer's Disease*.

[19] Harvey A. L., Young L. C., Viljoen A. M., Gericke N. P. (2011). Pharmacological actions of the South African medicinal and functional food plant *Sceletium tortuosum* and its principal alkaloids. *Journal of Ethnopharmacology*.

[20] Raheb H., Woodbury-Farina Y. B. M., Badmaev V. Translational study of standardized Zembrin extract and mesembrenone targeting PDE-4 
(phosphodiesterase subtype for regulation of cognition and mood).

[21] Nell H., Siebert M., Chellan P., Gericke N. (2013). A randomized, double-blind, parallel-group, placebo-controlled trial of extract *Sceletium Tortuosum* (Zembrin) in healthy adults. *Journal of Alternative and Complementary Medicine*.

[22] DSM-IV-TR American Psychiatric Association (2000). *Diagnostic and Statistical Manual of Mental Disorders*.

[23] Zimmerman M., Chelminski I., Posternak M. (2004). A review of studies of the Hamilton depression rating scale in healthy controls: implications for the definition of remission in treatment studies of depression. *Journal of Nervous and Mental Disease*.

[24] Gualtieri C. T., Johnson L. G. (2006). Reliability and validity of a computerized neurocognitive test battery, CNS vital signs. *Archives of Clinical Neuropsychology*.

[25] Martyr A., Clare L. (2012). Executive function and activities of daily living in Alzheimer's disease: a correlational meta-analysis. *Dementia and Geriatric Cognitive Disorders*.

[26] Albert M. S. (2011). Changes in cognition. *Neurobiology of Aging*.

[27] Marshall G. A., Rentz D. M., Frey M. T., Locascio J. J., Johnson K. A., Sperling R. A. (2011). Executive function and instrumental activities of daily living in mild cognitive impairment and Alzheimer's disease. *Alzheimer's and Dementia*.

[28] Clark L. R., Schiehser D. M., Weissberger G. H., Salmon D. P., Delis D. C., Bondi M. W. (2012). Specific measures of executive function predict cognitive decline in older adults. *Journal of the International Neuropsychological Society*.

[29] Allain P., Etcharry-Bouyx F., Verny C. (2013). Executive functions in clinical and preclinical Alzheimer's disease. *Revue Neurologique*.

[30] Braskie M. N., Thompson P. M. (2013). Understanding cognitive deficits in alzheimer's disease based on neuroimaging findings. *Trends in Cognitive Sciences*.

[31] Schroeter M. L., Vogt B., Frisch S., Becker G., Barthel H., Mueller K., Villringer A., Sabri O. (2012). Executive deficits are related to the inferior frontal junction in early dementia. *Brain*.

[32] Burgin A. B., Magnusson O. T., Singh J., Witte P., Staker B. L., Bjornsson J. M., Thorsteinsdottir M., Hrafnsdottir S., Hagen T., Kiselyov A. S., Stewart L. J., Gurney M. E. (2010). Design of phosphodiesterase 4D (PDE4D) allosteric modulators for enhancing cognition with improved safety. *Nature Biotechnology*.

[33] Gericke N., Viljoen A. M. (2008). Sceletium—a review update. *Journal of Ethnopharmacology*.

[34] Rutten K., Basile J. L., Prickaerts J., Blokland A., Vivian J. A. (2008). Selective PDE inhibitors rolipram and sildenafil improve object retrieval performance in adult cynomolgus macaques. *Psychopharmacology*.

[35] Hansen R. T., Zhang H.-T. (2013). Senescent-induced dysregulation of cAMP/CREB signaling and correlations with cognitive decline. *Brain Research*.

[36] Homberg J. R. (2012). Serotonin and decision making processes. *Neuroscience and Biobehavioral Reviews*.

[37] Sheline Y. I., West T., Yarasheski K., Swarm R., Jasielec M. S., Fisher J. R., Ficker W. D., Yan P., Xiong C., Frederiksen C., Grzelak M. V., Chott R., Bateman R. J., Morris J. C., Mintun M. A., Lee J.-M., Cirrito J. R. (2014). An antidepressant decreases CSF A*β* production in healthy individuals and in transgenic AD mice. *Science Translational Medicine*.

[38] Rodríguez J. J., Noristani H. N., Verkhratsky A. (2012). The serotonergic system in ageing and Alzheimer's disease. *Progress in Neurobiology*.

[39] Terburg D., Syal S., Rosenberger L. A., Heany S., Phillips N., Gericke N., Stein D. J., Van Honk J. (2013). Acute effects of sceletium tortuosum (Zembrin), a dual 5-HT reuptake and PDE4 inhibitor, in the human amygdala and its connection to the hypothalamus. *Neuropsychopharmacology*.

[40] Vecsey C. G., Baillie G. S., Jaganath D., Havekes R., Daniels A., Wimmer M., Huang T., Brown K. M., Li X.-Y., Descalzi G., Kim S. S., Chen T., Shang Y.-Z., Zhuo M., Houslay M. D., Abel T. (2009). Sleep deprivation impairs cAMP signalling in the hippocampus. *Nature*.

[41] Schaefer T. L., Braun A. A., Amos-Kroohs R. M., Williams M. T., Ostertag E., Vorhees C. V. (2012). A new model of Pde4d deficiency: genetic knock-down of PDE4D enzyme in rats produces an antidepressant phenotype without spatial cognitive effects. *Genes, Brain and Behavior*.

